# Operant Sensation Seeking Requires Metabotropic Glutamate Receptor 5 (mGluR5)

**DOI:** 10.1371/journal.pone.0015085

**Published:** 2010-11-30

**Authors:** Christopher M. Olsen, Daniel S. Childs, Gregg D. Stanwood, Danny G. Winder

**Affiliations:** 1 Department of Molecular Physiology and Biophysics, Vanderbilt University School of Medicine, Nashville, Tennessee, United States of America; 2 Center for Molecular Neuroscience, Vanderbilt University School of Medicine, Nashville, Tennessee, United States of America; 3 Vanderbilt Kennedy Center for Research on Human Development, Vanderbilt University School of Medicine, Nashville, Tennessee, United States of America; 4 Department of Pharmacology, Vanderbilt University School of Medicine, Nashville, Tennessee, United States of America; Medical College of Georgia, United States of America

## Abstract

Pharmacological and genetic studies have suggested that the metabotropic glutamate receptor 5 (mGluR5) is critically involved in mediating the reinforcing effects of drugs of abuse, but not food. The purpose of this study was to use mGluR5 knockout (KO), heterozygous (Het), and wildtype (WT) mice to determine if mGluR5 modulates operant sensation seeking (OSS), an operant task that uses varied sensory stimuli as a reinforcer. We found that mGluR5 KO mice had significantly reduced OSS responding relative to WT mice, while Het mice displayed a paradoxical increase in OSS responding. Neither KO nor Het mice exhibited altered operant responding for food as a reinforcer. Further, we assessed mGluR5 KO, Het and WT mice across a battery of cocaine locomotor, place preference and anxiety related tests. Although KO mice showed expected differences in some locomotor and anxiety measures, Het mice either exhibited no phenotype or an intermediate one. In total, these data demonstrate a key role for mGluR5 in OSS, indicating an important role for this receptor in reinforcement-based behavior.

## Introduction

Metabotropic glutamate receptor 5 (mGluR5) is a receptor that is thought to regulate anxiety and drug reward, though the mechanisms remain uncertain [1]–[6]. Pharmacological or genetic blockade of mGluR5 signaling attenuates self-administration of many classes of drugs of abuse, including psychostimulants, opiates, nicotine and ethanol [7]–[15]. Despite the widespread actions on drug self-administration, most studies have found that self-administration of food is unaffected by mGluR5 antagonism or deletion [7], [8], [15], [16] (although see [11], [17]). Despite the large number of studies investigating the role of mGluR5 in regulating intake of drugs of abuse, little effort has been made to advance our understanding of mGluR5 signaling on non-drug rewards beyond food.

We have recently described a method of assessing non-drug reinforcement by “self-administration” of varied sensory stimuli, a method we termed “operant sensation seeking” (OSS) [18], [19]. This procedure is based on a phenomenon whereby animals of several species will perform operant responses for visual and/or auditory stimuli [20]-[24]. We further found that OSS is sensitive to disruption of dopamine signaling in much the same manner as operant responding for psychostimulants [19]. This finding prompted us to examine another target critical for mediating drug reinforcement, mGluR5, on OSS. We hypothesized that mGluR5 knockout mice would have disrupted OSS performance based on a previous report that these mice do not acquire cocaine self-administration [7]. The present studies indicate a critical role for mGluR5 in OSS, suggesting that mGluR5 regulates non-drug reward processes. We next did a series of experiments to determine if other reward, anxiety, and locomotor phenotypes were affected by mGluR5 deletion or haploinsufficiency. It was predicted that these measures would also be influenced by mGluR5 disruption, as studies using pharmacological or genetic blockade of mGluR5 have revealed a role for mGluR5 in these phenotypes [2]–[4], [7], [25]. We found that mGluR5 heterozygous did not show a phenotype in any other measures and that alterations in locomotor or anxiety-related behavior do not appear to be responsible for the differences in OSS. In total these data suggest a unique role for mGluR5 in reinforcement-based behavior.

## Results

### Experiment 1: Operant Conditioning

**Figure 1:**
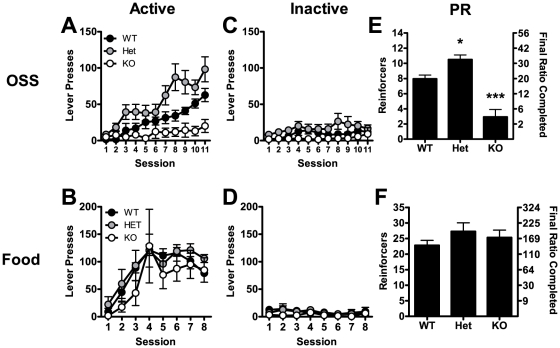
Operant sensation seeking (OSS) and operant responding for food on fixed and progressive ratio schedules. (A, B) Active lever pressing during 11 daily FR-1 OSS (A) or food (B) operant sessions. (C, D) Inactive lever pressing during the same sessions. (E, F) Progressive ratio responding following FR-1 sessions (bars represent mean of PR days 4 and 5± SEM). OSS: n = 8–12, Food: n = 5–6. *p<0.05, ***p<0.001.

**Figure 2:**
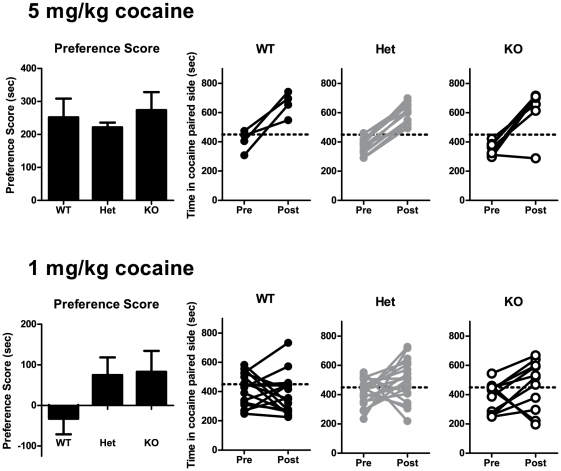
Place conditioning for cocaine. Preference scores (post-test minus pre-test time spent on cocaine-paired side) following place conditioning with 5 mg/kg (top) or 1 mg/kg (bottom) cocaine. Individual pre- and post-test values are shown for each genotype on the right of each preference graph. Bars represent mean ± SEM.

Analysis of FR-1 operant responding for OSS revealed a significant main effect of mGluR5 genotype on active lever responding for OSS (F(2,290) = 9.5, p<0.01), but not inactive lever responding (F(2,290) = 3.1, n.s.; Fig. 1A,C). There was also a significant main effect of session number in both active lever (F(10,290) = 17.1, p<0.0001) and inactive lever responding (F(10,290) = 2.3, p<0.05) in OSS, although an interaction effect was only observed for the active lever (F(20,290) = 3.4, p<0.0001). These data indicate that there was a significant influence of mGluR5 genotype on operant learning with OSS reinforcement on an FR-1 schedule of reinforcement, where heterozygous mice had enhanced responding and knockout mice had attenuated responding. Analysis of FR-1 operant responding for food reinforcer revealed no significant effect of mGluR5 genotype on either active (F(2,91) = 0.9, n.s.) or inactive lever pressing (F(2,91) = 1.1, n.s.), although there was a significant effect of session number on active (F(7,91) = 12.4, p<0.0001), but not inactive (F(7,91) = 1.1, n.s.) lever pressing (Fig. 1B,D). There was also no interaction effect of genotype and session number on active (F(14,91) = 0.4, n.s.) or inactive lever pressing (F(14,91) = 0.5, n.s.). Analysis of PR responding revealed a significant genotype effect on OSS (F(2,29) = 29.9, p<0.0001), but not food (F(2,13) = 0.44, n.s.; Fig. 2E,F). Multiple comparisons performed on OSS data revealed that heterozygous mice had a significant (p<0.05) elevation in responding relative to wildtypes, while knockout mice had dramatically blunted responding (p<0.001).

### Experiment 2: Cocaine Place Conditioning

To test another measure of reward-related behavior, we performed place conditioning with cocaine. First, a group of mice were conditioned using 5 mg/kg cocaine (Fig. 2). We found that all three genotypes of mice developed a similar degree of conditioned place preference to this dose (F(2,23) = 0.74, n.s.). To determine if mGluR5 heterozygous or knockout mice had enhanced sensitivity to the conditioned reinforcing effects of a low dose of cocaine, another group of mice was conditioned with 1 mg/kg cocaine. We found that neither heterozygous nor knockout mice developed a greater place preference to this low dose of cocaine than wildtype mice (F(2,43) = 2.1, n.s.).

### Experiment 3: Novel Open Field Activity and Locomotor Response to Cocaine

**Figure 3:**
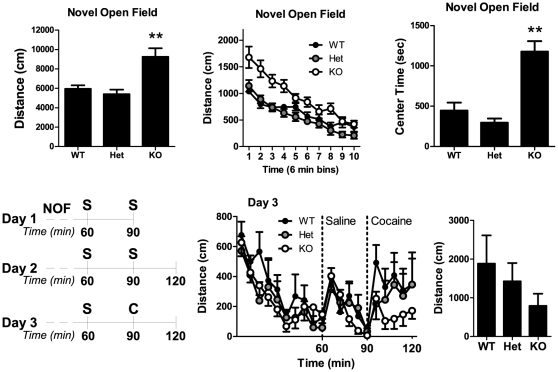
Locomotor response to novel environment and cocaine. (A) Total distance traveled and (B) distance in 6 min bins during a 60 min novel open field (NOF) test. (C) Center time during the NOF test. (D) Timeline of locomotor testing. Day 1 included the NOF test, then the first habituation injection of saline followed by 30 min more time in the open field. S denotes a saline injection, C denotes a cocaine injection (10 mg/kg i.p). (E) Locomotor data from the mice on day 3. Mice were placed into the open field chambers, allowed one hour to habituate, injected with saline at min 60, then with cocaine at min 90. (F) Distance traveled following cocaine injection (day 3, min 90–120). Bars and symbols represent mean ± SEM n = 6–9 per genotype. **p<0.01.

mGluR5 deletion has been reported to increase locomotor activity in a novel environment and strongly attenuate the locomotor activating effects of cocaine [7], [25]. We examined locomotor responses to two different stimuli: a novel environment and cocaine administration (Fig. 3). Consistent with previous data, we found a significant genotype effect on locomotor activity in the novel open field (F(2,28) = 10.7, p<0.001). Post hoc tests showed that KO mice had enhanced locomotor activity relative to WT (p<0.01), although Het mice did not differ from WTs. Analysis of novel open field data by 6 min time bins again revealed a significant genotype effect (F(2,279) = 12.8, p<0.001), as well as an effect of time (F(9,279) = 35.2, p<0.0001), and a significant genotype × time interaction (F(18,279) = 4.3, p<0.0001). To get a crude measure of anxiety-like behavior, we measured the time spent in a center zone (12.7×12.7 cm) during the novel open field test, as center time has been shown to increase following treatment with anxiolytic drugs [26]. Consistent with the mGluR5 receptor being critical in anxiety-like behavior, there was a significant effect of genotype on center time in the NOF (F(2,28) = 23.8, p<0.0001). Post hoc analysis revealed a profound increase in center time in knockout mice relative to wildtype (p<0.01), consistent with lower anxiety-like behavior in these mice.

Following NOF, a subset of mice were further habituated to the open field, then given an injection of cocaine (10 mg/kg, i.p., Fig. 3) and locomotor response was measured in the habituated open field. There was a trend for cocaine to have its greatest locomotor stimulating effect on wildtype mice, with decreasing effects on heterozygous and knockout mice.

### Experiment 4: Elevated Plus Maze, Light-Dark Exploration Test, and Modified Irwin Screen

**Figure 4:**
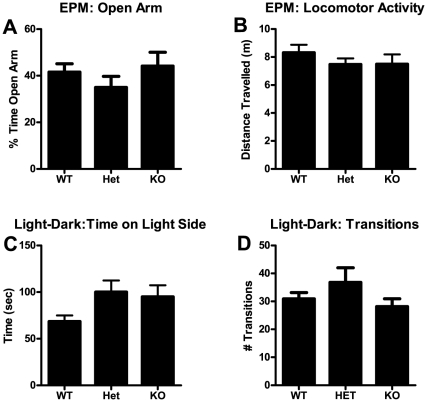
Anxiety-like Behavior as Measured by Elevated Plus Maze (EPM) and Light-Dark Exploration test. (A) Open arm time as a function of total arm time on the EPM. (B) Locomotor activity during the same test. (C) Time spent on the light side and (D) transitions between light and dark sides during a 5 min light-dark exploration test. Bars represent mean ± SEM. n = 9–13 per genotype.

To investigate anxiety related measures in mGluR5 heterozygous and knockout mice, we tested them in elevated plus maze (EPM) and the light-dark exploration test. Considering the previously established anxiolytic phenotype of mGluR5 null mutant mice, EPM and light-dark exploration testing were conducted under low lighting conditions to enhance our ability to detect a potential anxiogenic phenotype in the heterozygous mice (Fig. 4). We found no significant effect of genotype in the EPM as measured by percent of open arm time relative to total arm time (F(2,31) = 0.95, n.s.). Unlike the NOF, however, we saw no difference in locomotor activity in the testing apparatus that mice were naïve to (F(2,31) = 0.61, n.s.), suggesting that general differences in locomotor activity did not confound our measures. Similar to results from EPM, we saw no significant differences between genotypes in the light-dark exploration test as measured by time spent in the light chamber (F(2,31) = 1.9, n.s.) or in the number of transitions between chambers (F(2,31) = 1.4, n.s.). At least two weeks following the light-dark test, mice were tested for physiological and reflexive measures using a modified Irwin battery (29). Two independent observers conducted this battery, and scores represent the average of two observations. The modified Irwin screen indicated that the three mGluR5 genotypes were similar in most measures (Table 1). One difference between genotypes was in body weight (F(2,30) = 5.0, p<0.05), whereby knockouts (p<0.05), but not heterozygous, weighed less than wildtype mice. This difference has been reported previously and has been attributed to alterations in appetite and energy balance induced by null deletion of the mGluR5 receptor [27].

**Table 1:** MODIFIED IRWIN.

	+/+	+/−	−/−
*Physical condition*			
Body weight (g)	23.4±0.62	23.6±0.91	20.5±0.73*
Rectal temperature (°C)	37.2±0.16	36.9±0.15	37.0±0.18
Normal presence of whiskers (%)	100	91.7	83.3
Well groomed (%)	100	100	100
Piloerection (%)	0	0	0
Fur missing on face (%)	5.6	0	4.2
Fur missing on body (%)	11.1	0	0
Wounds (%)	0	0	0
*Behavior in novel environment*			
Normal transfer behavior (%)	100	100	95.8
Normal body positioning (%)	100	100	100
Spontaneous activity (%)	0	0	0
Normal respiration rate (%)	100	100	95.8
Tremors (%)	0	0	0
Palpebral closure (%)	0	0	0
Piloerection (%)	0	0	0
Normal gait (%)	100	100	100
*Reflexes or Reaction to Stimuli*			
Touch escape (%)	100	100	100
Positional passivity (%)	0	0	0
Trunk curl (%)	100	95.8	100
Reaching reflex Before vibrissae contact (%) Upon vibrissae contact (%) None (%)	10000	91.78.30	10000
Normal body tone (%)	100	100	100
Pinna reflex (%)	100	91.7	100
Preyer reflex at ∼90 dB (%)	72.2	83.3	75
Toe pinch response (%)	88.9	91.7	91.7
Righting reflex (%)	100	100	100
Air righting reflex (%)	100	100	100
Wire hang latency to fall (s)	28.9±7.1	24.2±4.3	26.8±3.9
*Measures during supine restraint*			
Skin Color- pink (%)	88.9	95.8	100
Normal heart rate (%)	100	100	100
Normal limb tone (%)	100	100	100
Normal abdominal tone (%)	100	100	100
*Provoked biting response*Normal (%)No response (%)	94.55.5	95.64.4	66.725
Over aggressive (%)	0	0	8.3
Excess salivation (%)	0	0	0

Modified Irwin Screen: Mice were screened in a battery of measures to assess overall health and basic sensory and reflexive function. Values reflect the mean ± SEM or the percentage of animals exhibiting each attribute unless noted. Scores are the mean of observations conducted by 2 different experimenters. Sample size: wildtype (+/+) n = 9, heterozygous (+/−) n = 12, knockout (−/−) n = 12.

## Discussion

We have previously demonstrated that C57Bl/6J mice self-administer varied visual and auditory cues in operant sessions without any prior training, a phenomenon we term “operant sensation seeking” (OSS) [18], [19]. OSS is dependent on dopamine signaling, as it is altered by the dopamine antagonist cis-flupenthixol, and D1 knockout mice fail to acquire OSS despite being able to acquire food self-administration [19], [28]. D1 knockout mice also fail to self-administer cocaine [28], suggesting that the reinforcing effects of OSS may be mediated by neural substrates more similar to psychostimulants than to food. We tested the hypothesis that another receptor important in mediating the effects of drugs of abuse, mGluR5, mediates the reinforcing effects of OSS using mGluR5 heterozygous and knockout mice. We found that, like D1 deletion, mGluR5 deletion nearly abolished operant responding for sensory stimuli. To determine if D1 levels were altered in mGluR5 Het and KO mice, tissue from the nucleus accumbens (NAc) of naïve mice was taken and probed for levels of D1 protein. We found that there was no difference in NAc D1 levels between any of the mGluR5 genotypes (Supporting Information S1), suggesting that changes in NAc D1 content did not contribute to the phenotypes observed in OSS. Under the same conditions, separate mGluR5 KO mice were able to acquire operant responding for food, indicating that the loss of mGluR5 did not have non-specific effects on operant learning. This is an important finding, as mGluR5 knockout mice have been previously shown to fail to self-administer cocaine [7]. The present data combined with our previous finding that dopamine D1 receptor knockout mice fail to acquire OSS [19] suggests that OSS shares neural mechanisms with those mediating psychostimulant self-administration. We further demonstrated that mGluR5 heterozygous mice had a paradoxical increase in OSS responding. This is a unique finding, but it is not unprecedented. For example, dopamine transporter (DAT) KO mice have attenuated novelty seeking in some measures, while DAT Het mice have a paradoxical increase in novelty seeking [29]. The underlying cause for our findings is unclear, but altered OSS behavior in both mGluR5 KO and Het mice suggests that even subtle variation in mGluR5 expression is capable of altering reinforcement behavior in this paradigm.

A previous report found that mGluR5 KO mice failed to self-administer cocaine, although Het mice were not investigated [7]. To determine if mGluR5 KO or Het mice had altered sensitivity to the conditioned reinforcing effects of a psychostimulant, we performed cocaine place conditioning experiments. We found that mice would develop a similar degree of place preference for 5 mg/kg cocaine regardless of mGluR5 genotype, while no genotypes showed a preference for 1 mg/kg. These results are in contrast to a previous report that mGluR5 deletion abolished the acute reinforcing effects of cocaine [7]. One major difference between the previous study and this one is the different tests to measure cocaine reinforcement. The prior report was based on self-administration, while our data is based on place conditioning. Although there are several differences between these tests, one major difference lies in the measurement of acute (self-administration) versus conditioned (place conditioning) reinforcement. A similar discrepancy between these two tests has been noted before in mGluR5 knockout mice in response to ethanol [30]. Other studies using dopamine D1 receptor knockout mice in drug self-administration and place conditioning have also produced conflicting results [28], [31].

A recent paper identified a role for the mGluR5 receptor in Pavlovian to instrumental transfer; that is, when mGluR5 was blocked during Pavlovian conditioning training, the cue later failed to serve as a reinforcer in an operant test of conditioned reinforcement [32]. Importantly, the authors found that mGluR5 antagonist given immediately prior to testing had no effect on conditioned reinforcement. This suggests that, although mGluR5 transmission is critical for the establishment of incentive motivational properties of a cue stimulus (i.e. auditory or visual cue), it does not appear to interfere with the acute conditioned reinforcement or perception of the cue stimuli. Additionally, the mGluR5 antagonists MPEP and MTEP have been shown to interfere with drug self-administration and reinstatement in the absence of discrete cues [14], demonstrating that drug reinforcement and seeking behaviors can be attenuated by mGluR5 antagonists independently of any actions on discrete cue stimuli.

It is important to consider potential differences in locomotor activity that could influence phenotypes such as OSS. To determine if altered mGluR5 expression levels also affected locomotor activity relevant to OSS, we tested locomotor activity in a novel environment and after habituation. Following habituation, we also tested the locomotor sensitivity to cocaine. mGluR5 deletion has been reported to increase novelty-induced locomotor activity [25] and block the locomotor activating effects of cocaine [7] (although see [25]). We found that only mGluR5 knockout mice were hypersensitive to the locomotor stimulating effects of a novel environment, although all mice had similar locomotor activity after habituation. These findings are not consistent with increased OSS responding exhibited by Hets or depressed OSS responding by KOs. We also found that in a habituated environment, KO and Het mice had a reduced locomotor response to cocaine relative to wildtype mice. Consistent with our data measuring operant responding for food (Fig. 1B,D,F), locomotor measures indicate that there were no general differences in locomotor or exploratory behavior that were responsible for the genotype differences in OSS.

mGluR5 has been strongly implicated in mediating anxiety behavior in rodent models [4], [33], [34], and clinical trials with the mGluR5 antagonist fenobam have supported this idea [35], [36]. Our results showing increased NOF center time in mGluR5 knockout mice are consistent with this and expand on a report demonstrating reduced anxiety-like behavior in these mice [34]. When tested using other measures of anxiety-like behavior, KO mice did not show reduced anxiety-like behavior. This is likely due to our conditions of testing. In both the elevated plus maze and the light-dark exploration test, light levels were very low (see methods for details) in an attempt to maximize sensitivity for detecting potential anxiogenic effects of mGluR5 haploinsufficiency (we hypothesized that anxiety-related phenotypes may track with the OSS data). The expected reduction of anxiety-like behavior induced by low light conditions is especially apparent in the EPM, as WT mice had very high levels of open arm time (over 40% of total arm time), which likely lead to a ceiling effect in this measure.

A modified Irwin screen indicated that in most regards, there were no differences between mGluR5 genotypes. Among these tests was the reaching reflex, which assesses a mouse's ability to reach toward an edge using a visual cue. Over 90% of all mice were able to reach prior to vibrissae contact, indicating that there are no gross alterations in vision in any of the genotypes. A notable finding in the Irwin screen was the decreased body weight observed in mGluR5 knockout mice. This difference has been reported previously and was attributed to alterations in appetite induced by mGluR5 deletion [27]. We found no difference between genotypes in operant responding for food reward (Figs. 1B & D), suggesting that food still serves as a reinforcer in mGluR5 knockout mice, and that under the current conditions, knockout mice will exert a similar level of effort to obtain food reinforcer (Fig. 1F). We also found no difference in rectal temperature between genotypes (Table 1), suggesting that there are no gross metabolic disturbances induced by disruption of grm5.

In conclusion, we found that OSS was highly susceptible to changes in mGluR5, as both the Hets and KOs had altered responding. We also found that different traits associated with the mGluR5 receptor are differentially vulnerable to variance in the grm5 gene. Unlike OSS, haploinsufficiency of the grm5 gene did not significantly affect anxiety-like behavior. Similarly, we found that conditioned place preference for cocaine was similar across genotypes for 5 mg/kg, although there was a trend for Het and KO to have greater preference for 1 mg/kg. Considering that variation in the human mGluR5 gene (GRM5) has been linked to alcohol dependence [37], the present findings may provide additional insight to the relationship between GRM5 variation and its influence on traits such as anxiety and sensation seeking. The data presented also expand on previous work [19] that suggests that reinforcing sensory stimuli engage similar neural mechanisms to psychostimulants.

## Materials and Methods

### Ethics Statement

Protocols were performed in compliance with the Guide for the Care and Use of Laboratory Animals (NIH, Publications 865–23) and were approved by the Institutional Animal Care and Use Committee, Vanderbilt University (protocols M/05/063, M/09/126, and M/09/152).

### Animal Care

mGluR5 (*grm5*) null mutant, heterozygous, and wildtype littermate mice [38], [39] were bred in house from heterozygous matings of animals backcrossed onto C57Bl/6J background >5 generations and genotypes were determined by PCR analysis of tail tissue using standard procedures. Male littermate mice were housed 2–5 per cage in a temperature and humidity controlled environment (lights on 0600–1800 h) within AAALAC approved Vanderbilt University Animal Care Facilities. Cages contained corn cob bedding supplemented with a small amount of cellulose bedding (Carefresh®). Food and water were available *ad libitum* unless noted. Mice were handled for at least three days prior to the beginning of experiments. All procedures were approved by the Animal Care and Use Committee at Vanderbilt University.

### Experiment 1: Operant Conditioning

48 male mGluR5 null mutant, heterozygous, and wildtype littermate mice underwent operant conditioning in either a food self-administration task or operant sensation seeking [18], [19]. Operant sessions were conducted between 0900–1400 h. Operant training chambers were as described [40]–[42], with levers mounted 2.2 cm above the grid floor and cue lamps (yellow LEDs) mounted 2 cm above them. At the beginning of each session, the house light and exhaust fan were turned on, and both levers were extended. Each mouse was assigned either the left or right lever to the active lever, and the side of the active lever was counterbalanced between mice within each genotype. In the operant sensation seeking (OSS) group, a compound visual/auditory stimulus was presented after completion of the required ratio on the active lever, while presses on the inactive lever were counted but had no programmed consequence. The compound stimulus was the same as previously described [18], [19] and consisted of flashing cue lights (random duration of 2, 4, 6, or 8 sec; random flash rate of 0.625, 1.25, 2.5 or 5 Hz), with each flash randomly on the right or left side of the chamber and the house light was turned off during the visual stimuli. The auditory stimulus was activation of an infusion pump located within the cubicle (no infusion is made). In the food self-administration group, completion of the required ratio on the active lever resulted in illumination of the cue lights and elevation of a dipper cup containing ∼40 ul of liquid food reinforcer (25% Vanilla Ensure®). The dipper was available for 10 seconds after head entry into the dispenser, and the cue lights remained illuminated until the end of the 10-second access. Separate mice were used for food self-administration and OSS. Mice in both the OSS and food groups were food restricted (chow available ∼6 hours per day following operant sessions) for the duration of operant experiments. The experiment began with one-hour sessions where each active lever press was reinforced (Fixed Ratio-1, FR-1). Following 11–14 (OSS) or 8 (food self-administration) sessions on the FR-1 schedule, mice responded on a progressive ratio (PR) schedule of reinforcement for five additional days in two-hour sessions. No exclusion criteria based on operant performance were set. Within each PR session, the schedule of reinforcement was increased in the following pattern: 1, 2, 4, 6, 9, 12, 16, 20, 25, 30, etc. [40], [43]. The mean number of reinforcers on the final two days was measured for each animal.

### Experiment 2: Cocaine Place Conditioning

72 male mice were divided into two groups and tested using either 1 or 5 mg/kg cocaine. Place conditioning experiments were conducted in commercially available two-chamber conditioning apparatuses (ENV-512 inserts for ENV-510 chambers, MED Associates) housed individually within sound-attenuating chambers (MED Associates ENV-515). Each chamber of the apparatus was identical with the exception of the floor material (bar floor vs. wire mesh floor). These floor materials have previously been described as imparting different preferences in mice [44], and our own pilot data has indicated that these chambers are biased, therefore experiments were performed in a biased manner. In all mice, cocaine was paired with the less preferred side (bar floor) and saline was paired with the preferred side (mesh floor). On the first day of the experiment, mice were tested in an initial 15 min pre-conditioning session in which they had access to both chambers (testing began at 1200 h). During days 2–5, mice were conditioned twice per day in 15 min sessions spaced four hours apart (session 1∶1000 h; session 2∶1400 h). In the conditioning sessions, mice were either injected with saline and immediately confined to the grid chamber or injected with cocaine and immediately confined to the bar chamber. Within each genotype, mice were randomly assigned which type of conditioning session to begin with (saline or cocaine). For all animals, the conditioning sessions were alternated such that the first session of the day was different than the first session of the previous day. On day 6, a 15 min post-conditioning test was conducted in which animals had free access to both chambers.

### Experiment 3: Novel Open Field Activity and Cocaine-Induced Locomotor Activity

31 male mice were tested for open field locomotor activity in a novel environment as previously described [40]. After one-hour acclimation, mice were tested in a one-hour session using automated experimental chambers (27×27 cm; MED-OFA-510; MED Associates, St. Albans, VT) within sound attenuating chambers. Analysis was performed using Activity Monitor v5.10 (MED Associates). A subset of mice was tested further to determine cocaine induced locomotor activity. Following the novel open field (NOF) test, these mice received their first habituation injection of saline (5 ml/kg, i.p.) and were placed back into the open field chamber for an additional 30 min. The next day, mice were placed in the chambers for one hour, and then received two saline injections 30 min apart while remaining in the chambers. On the final day (day 3), mice were placed into the open field chambers for one hour, received a saline injection, then received an injection of cocaine (10 mg/kg, 5 ml/kg, i.p.) 30 min later. Mice remained in the open field chambers for 30 min following cocaine and locomotor activity was recorded.

### Experiment 4: Elevated Plus Maze, Light-Dark Exploration Test, and Modified Irwin Screen

#### Elevated plus maze

34 male mice were tested on the elevated plus maze followed by the light-dark exploration test, then subjected to a modified Irwin screen. The maze consisted of two white closed arms protected by black acrylic walls 15 cm tall, bisected by two white open arms with raised edges and was lit by incandescent lighting (9–11 lux per arm). Sessions were recorded by a ceiling-mounted video camera connected to a computer using video acquisition and analysis software (Anymaze, Stoelting). Mice were allowed one hour to acclimate after being transported to the experimental room. Experimental sessions were five minutes in duration, and the apparatus was cleaned with 30% ethanol between each trial. Data were represented as % open arm time divided by total arm time (i.e., time not in the center zone). Locomotor activity was represented as distance traveled throughout the entire maze.

#### Light-Dark Test

48 hours following elevated plus maze testing, mice were tested in the light-dark exploration test. Eight light-dark apparatuses consisted of inserts (ENV-511, Med Associates) placed into the open field chambers described in Experiment 3. The lighting was ∼30 lux in the bright chamber and ∼2 lux in the dark chamber. After transport to the experimental room, mice were given one hour to acclimate before testing. Each test was 5 minutes and began by placing the mouse into the light chamber facing away from the entrance to the dark chamber. Latency to enter the dark side, time spent in each chamber, and the number of transitions was measured.

#### Modified Irwin Screen

At least one week following the light-dark exploration test, mice were screened for physiological and reflexive measures in a modified Irwin battery [45] using a three point scale similar to that previously described [46]. Mice were scored by the mean of two independent observers. Percentage of mice within a category was calculated as the proportion of mice meeting the specified category. In the instance of discrepancy between the two observers, each mouse was counted as a half being in that category.

### Statistical Analysis

Genotype differences were compared by between-subjects ANOVA followed by Dunnett's multiple comparisons tests when significant overall ANOVA effects were found. In the instance that multiple sessions were also performed on each subject, two-way repeated measures ANOVA was performed with session as the repeated factor. In the case of heterogeneous variance, Games-Howell multiple comparisons were employed. For the modified Irwin screen, measurements that were not done on a three-point scale were compared by one-way ANOVA (genotype) followed by Dunnett's *post hoc* testing comparing each genotype to wildtype.

## Supporting Information

Supporting Information S1 Methodology for and Measurement of D1 and mGluR5. Levels of D1 (A) and mGluR5 (B) receptor protein in nucleus accumbens of naïve mGluR5 WT, Het, and KO mice. Data analyzed by 1 way ANOVA followed by Dunnet's *post hoc* tests comparing Het and KO to WT. N = 3-7, **p<0.01.
(PDF)
