# Supplementary material for: Operant Sensation Seeking Requires Metabotropic Glutamate Receptor 5 (mGluR5)
Source: PLoS One. 2010 Nov 30;5(11):e15085. doi: 10.1371/journal.pone.0015085 (PMC2994905; doi:10.1371/journal.pone.0015085)
Supplement: Supporting Information S1 — Methodology for and Measurement of D1 and mGluR5. Levels of D1 (A) and mGluR5 (B) receptor protein in nucleus accumbens of naïve mGluR5 WT, Het, and KO mice. Data analyzed by 1 way ANOVA followed by Dunnet's post hoc tests comparing Het and KO to WT. N = 3-7, **p<0.01. (PDF) [file pone.0015085.s001.pdf]

Supplemental Material

Methods

Naïve male mGluR5 WT, Het, and KO mice were injected with Nembutal (150 mg/kg, i.p.) and rapidly decapitated. Brains were placed into a chilled matrix (ASI Instruments, Warren, MI) and 1 mm sections containing nucleus accumbens (NAc) were made and placed onto a Petri dish. The Petri dish was placed on top of a chilled aluminum block, and tissue was brought to a semifrozen state before punches were taken. Bilateral punches (0.50 mm; Fine Science Tools, Foster City, CA) were taken from slices containing NAc, collected in microcentrifuge tubes and stored at -80° C.

For immunoblotting, NAc samples were homogenized by sonication in 50 mL 2% SDS containing commercial protease and phosphatase inhibitors (Sigma Aldrich, P2714, P0044 and P5726). This crude homogenate was spun at RT for 15 min at 1000g and the supernatant was retained for Western blotting. Protein levels were determined by BCA protein assay (Thermo, #23225, Rockford IL). Equal protein concentrations were mixed with sample buffer and incubated at 65°C for 10 min prior to loading. Proteins were separated within 10% polyacrylamide gels and transferred to nitrocellulose membranes (0.45 mm). Membranes were blocked using 4% Blotto and 0.05% Tween-20 in PBS. Dopamine D1 receptors were probed using a rat anti-D1 monoclonal antibody (Sigma Aldrich, D-187, 1:250), and ECL detection (GE Healthcare, RPN2106). For anti-D1 probing, Tween-20 was increased to 0.1%. mGluR5 receptors were assayed using a rabbit polyclonal antisera (Millipore, MAB5675, 1:2500). Protein loading was verified by probing with a mouse anti-GAPDH antibody (Ambion, AM4300, 1:125,000). Primary antibodies were incubated overnight at 4° degrees and species-appropriate secondary antibodies were incubated for 60 min at RT (1:5,000-10,000). Immunoreactivity on the membranes was visualized using a Fuji LAS 4000 imaging station. Images were captured using a cooled CCD camera under "super sensitivity" settings. Images were converted to 8 bit gray scale and band densities were quantified using Image J. Optical density measures of D1 and mGluR5 were then corrected by measuring the optical density of GAPDH for each corresponding sample to determine a correction factor. The correction factor was calculated by converting each GAPDH band to a percentage of the mean GAPDH intensity then taking the inverse of this percentage. Each D1 and mGluR5 band density was multiplied by the corresponding correction factor to obtain the corrected optical density.

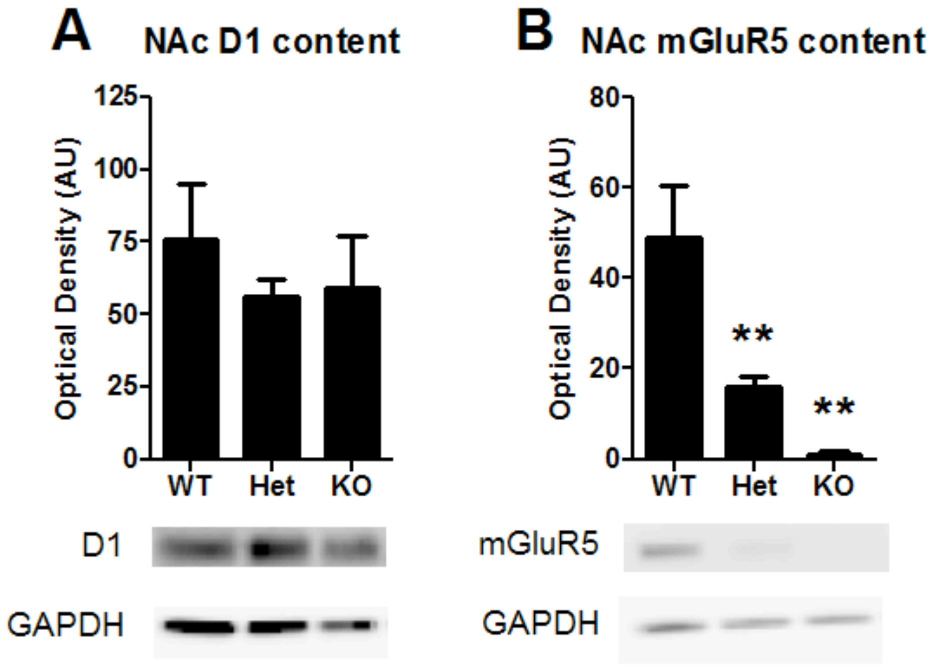
A
NAc D1 content
Optical Density (AU)
125
100
75
50
25
0
WT
Het
KO
D1
GAPDH
B
NAc mGluR5 content
Optical Density (AU)
80
60
40
20
0
**
**
WT
Het
KO
mGluR5
GAPDH
